# Idealism as an educational philosophy of mathematics teachers in Al Ain City Schools of the United Arab Emirates

**DOI:** 10.1371/journal.pone.0279576

**Published:** 2023-02-21

**Authors:** Omar M. Khasawneh, Adeeb M. Jarrah, Mohammad S. Bani Hani, Shashidhar Belbase

**Affiliations:** 1 College of Education, Yarmouk University-Irbid, Irbid, Jordan; 2 Department of Curriculum and Instruction, Emirates College for Advanced Education, Khalifa City, Abu Dhabi, United Arab Emirates (UAE); 3 Department of Curriculum and Instruction, College of Education, United Arab Emirates University, Al Ain, United Arab Emirates (UAE); University of Lisbon: Universidade de Lisboa, PORTUGAL

## Abstract

Educational philosophy, in general, is at the heart of the growth of education. It outlines the institution’s goals, subject matters, teaching methods, roles of teachers as well as the role of students, assessment methods, and teaching/learning experiences. The study aimed to identify the educational philosophical implications of idealism in schools in Al Ain city of the United Arab Emirates from the perspectives of mathematics teachers. The researchers used a questionnaire with thirty-two Likert-type items as a quantitative method for data collection. The instrument was administered to a randomly selected sample of 82 (46 male and 36 female) mathematics teachers in Al Ain city. The data were analyzed in IBM SPSS version 28 for one sample t-tests and independent samples t-tests to compare teachers’ perceptions of curriculum, education values, school functions, roles of teachers, and teaching methods with gender and school type. Further analyses included a one-way ANOVA for teaching experiences and teaching cycles, bivariate correlations between the variables, and a generalized linear model to identify the significant predictors of the teaching method. The findings of the study showed that mathematics teachers in Al Ain city embrace an idealistic philosophy of curriculum, educational values, the role of schools and teachers, and teaching methods in general. The teachers’ perceptions of the curriculum and school functions were found to be significant predictors of their teaching methods. These findings have both pedagogical and curricular implications.

## Introduction

There is a dominance of a specific educational philosophy in any industrialized nation in the logic of governing and leading over its present and future dogmatic sociology [1]. Thus, the purpose of this study is to determine the educational philosophical implications of Idealism throughout schools in Al Ain city in the United Arab Emirates from the perspectives of teachers. A particular philosophy, such as idealism for teaching, may help mathematics teachers enrich or improve teaching and implementing school curricula [2]. Idealism is an educational philosophy that focuses on the idea that "…pupil lives in an idea-centered mathematical world, but not an objective real world" [2] (p. 1).

Idealism, as an ancient philosophy known to humankind, has influenced the minds of men and women in the past and in contemporary times, even though societies at present times may not be motivated to follow any dogmatic beliefs or theories [3]. Nevertheless, Idealism has influenced many educators with self-reflection on consciousness and the inner dynamic phenomenon of the mind [4]. The influence of Idealism on education has gone a long way towards limiting some radical philosophies and creating the value of everlasting ideals and morals of existence [5–7].

As the first known school of philosophy, Idealism teaches that an idealist is a thinker who admires the mental state of human beings and has no concerns about the physical principles of life [8, 9]. Philosophically speaking, Idealism is derived from the term—the ideal, which stands for the completed practice of an idea or ideas [10, 11], and something created by mind or mind-dependent entity [12]. However, an idea means true and affirmed awareness [13]. Idealism is a method that stresses the pre-eminent importance of spirit, soul, or mind [10, 11, 14, 15].

The spirit, soul, or mind is the primary source of human understanding and the most important human organ [14]. The school curriculum must focus on the ideals that form the ultimate goal in education and life [10]. The school curriculum taught to pupils must provide subject matters that should be kept constant for all [16]. The school views facts perceived by the human mind through reasoning as more accurate than direct sensual experience [11]. However, the senses are no less important than the mind in terms of understanding [11]. The role of a school is to transfer knowledge from one generation to another [17]. The school views knowledge as an independent entity far from sensual experience and views subject matter as the core curriculum that must be the teachers’ focus [17].

A teacher, according to idealism, ought to focus on curricular activities that are part of the school curriculum. The teacher is the central core of the educational process [3]. The school maintains popular culture through teaching [18]. Educational objectives concentrate on exercising the human mind while ignoring physical entities, promoting idealistic thinking of the metaphysical world [16], and the intellectual growth of an individual [19]. The teacher focuses on brainstorming to extract ideas and meanings from students’ minds through discussions and dialogue [18]. The teacher is the ideal role model for his/her students, mentally as well as morally, to construct knowledge of the mathematical world as mind-dependent [18–21]. School motivates learners to become cooperative, obedient, and respectful of others [20]. The school works on implementing suggestions and instructions. All students study the same courses within the school. The relationship between teachers and students is looked upon as an official rapport perceived as concerned with teaching methods such as respecting spiritual and individual values through studying the local environment and subjects such as philosophy, history, the arts, and mathematics [10, 17, 21].

Philosophy, history, the arts, and mathematics are considered the primary subject matters the school offers [17]. Mathematics is the subject matter that schools offer in order to educate the human mind [10]. Extracurricular activities, such as school clubs and classroom activities are taken into account by the school. Teachers use lecturing as a teaching method to transmit accurate information to their pupils, which helps them store definite facts in their minds [17]. Teachers use such teaching methods as dialogue, discussions, and mental activities in order to solve problems [21]. Individual differences are taken into consideration by the school, which ought to employ a variety of teaching methods[10, 17, 21, 22].

School uses such teaching methods as analyzing as well as synthesizing information in order to solve problems [21]. Teachers evaluate their students in light of accurate measurements governed by the governing body, which is the Ministry of Education or education authorities, and in light of accurate measurements governed by the teachers themselves. According to idealistic educational thought, official examinations are the best way to measure students’ achievements [3]. This study was intended to identify the educational philosophical implications of Idealism throughout schools in the United Arab Emirates from mathematics teachers’ perspectives. Several studies on problem elements, such as casual observations, practical experience of the researchers, and insufficient previous research studies pertinent to the topic of concern led the researchers to determine the study problem.

### The study problem

’Mathematics Idealism,’ according to Ediger [2], could contribute to mathematics teachers and help in selecting "…objectives, learning opportunities, and evaluation procedures for pupils" [2] (p. 3). Since the foundations of study problems could be extracted from the scholastic interests of the researchers, preceding research studies, and literature, the problem of this study has been amplified due to several drives and motives. Such motives are the casual observations of the researchers, inferences from educational theories, previous studies’ recommendations, relevant literature, practical situations, and the field experiences of the researchers or experienced educators.

Experienced educators should be attentive to why and how school teachers reflect and perform in their classroom situations. This study stems from the researchers’ curiosity about implementing idealistic philosophical standards from the perspectives of mathematics teachers in schools in a city in the United Arab Emirates. Additionally, the insufficient research studies vis-à-vis this topic of concern, in addition to the teachers’ unacquaintedness with and elusiveness of an educational philosophy upon which they base their teaching, had been another motive for the researchers.

Driven by determination, the researchers also concluded that their college students possess limited knowledge regarding the connection between Idealism and mathematics education. Upon graduation, these students lack knowledge about the importance of mathematical connections to Idealism in teaching and learning due to an inadequate understanding of such connections. Therefore, this unfamiliarity with the connection between the philosophical view of idealism and mathematics teaching and learning is, in the researchers’ estimation, an issue worth studying because it is considered an accruing phenomenon among mathematics teachers in the United Arab Emirates and elsewhere.

In the United Arab Emirates, upon reviewing previous literature and research studies, the researchers conducted this study to identify and justify the educational implications of Idealism throughout schools in Al Ain city of the United Arab Emirates from the perspectives of mathematics teachers. Accordingly, this study is an effort to react to the consequent study questions. The research questions for this study were: (1) To what extent do mathematics teachers believe that the educational philosophical implications of Idealism as an educational theory throughout schools in the United Arab Emirates are implemented? (2) Are there any significant statistical differences between mathematics teachers’ responses due to their gender, type of school, years of experience, and school cycle on all questionnaire items? (3) Is there any significant correlation between teachers’ opinions about the curriculum, educational values, school functions, roles of teachers, and teaching methods to each other? (4) Is there any significant impact of gender, school type, years of experience, cycle of teaching, perceptions of curriculum, value of education, school function, and role of teachers on teaching methods?

In order to implement the idealistic educational approach in the Emirati educational system, the findings of this study may generate awareness of potential teaching methods in classrooms, and this study may be a significant piece of literature in reporting an authentic argument vis-à-vis this particular topic of concern. Plato’s Academy entrance is believed to have been written: *"Let no one ignorant of geometry enter here"* [23, 24]. The value of this study may be established as an occasion to construct bonds between idealist educational principles and mathematics teachers in the United Arab Emirates.

### Literature review

As an ancient philosophical movement, Idealism is a system concentrating on the importance of the human soul. The philosophy of Socrates (469–399 BC) and Plato (427–347 BC), whose essential beliefs focused on the human spirit as the most fundamental component, envisioned the world as an immaterial creation. Idealism, as an educational belief, describes learning as a remembrance; that is, people accumulate what exhibits that their spirits had been in the metaphysical world before arriving at the human body. Idealism’s central standpoint looks at the human soul as the main constituent of human beings. On the other hand, the world is regarded as nonmaterial in its absolute nature, upon which almost all Idealist philosophers settle [15]. In Idealism, reality is a mental thought, and experience is a consequence of physical capacities. The individual cannot distinguish between probing for the existence of things without the mental component [11, 22], as Idealism preaches in its form of educational philosophy.

Plato’s Academy entrance is believed to have been written: "Let no one ignorant of geometry enter here" or "no one is to enter unless they know mathematics" [23, 24]. Plato’s testimonial stresses the importance of mathematical Idealism. Mathematics Idealism, as an ancient philosophy, could help teachers decide on learning objectives and opportunities, besides the assessment measures for students. Plato (427–347 BC), whose philosophical thought affirms that the mind or soul is superior to the physique or body, promoted Idealism as an educational philosophy in Athens [25, 26]. Therefore, a solid educational program of study in mathematics ought to be provided to school students that is hinged on the philosophy of idealism. Learning mathematics helps pupils in terms of their academic achievement, because the soul/spirit rules the body, resulting in greater stages of selection, whereas the body takes people to poorer or lesser stages of selection and conclusions [25–27]. Ediger [25] further stated:

[T]he mathematics teacher needs to stress pupils attaining abstract content in mathematics, since this will aid mental development. Higher cognitive level objectives need to be selected and implemented in the mathematics curriculum…. Idealism stresses that pupils live in an idea-centered mathematical world, but not an objective real world. The mental development of the pupils is the number one goal of instruction. The mathematics curriculum is viewed here as a part of the general education curriculum [2] (pp. 4 & 18).

The general education curriculum is purposefully designed around the consolidating values and necessary thoughts of the field and delivers prospects for thorough consideration in a multiplicity of situations [10]. This kind of curriculum highlights systematic comprehension over the extent of analysis [3]. Mathematics, which offers an actual technique for constructing rational discipline based on reasoning [28], and inspires reasonable thinking plus mental objectivity, is a fundamental part of human thought and logic and is essential to our efforts to understand the world as well as ourselves [18]. Knowledge of mathematics plays an essential role in understanding the contents of additional school subject matters such as social studies, art, economics, and science. Geometry and Algebra were two major liberal arts in the Greek and medieval eras [29].

### Educational philosophical implications of idealism

A perfect triangle, described by mathematicians, would be an example of a description of the form or idea. Plato believed that those forms occur in a mental state, liberated from human minds. He believed that consistent knowledge existed among individuals who could realize truths beyond the world of ordinary experience. Those individuals must go through a challenging education in order to recognize the world of forms. For Plato, only exceptional individuals could rule frankly, arguing that the ideal ruler must first be a philosopher because philosophers can only recognize forms and ideas [30–32].

Plato tried his best to change the way people think worldwide regarding everything, including mathematics, ethics, and logic [33]. The Theory of Forms is one of his most significant contributions to philosophy. This theory affirms that the physical world is not real, and the ultimate reality is beyond this physical world [31]. Plato discussed this theory in different dialogues; the most well-known of his works was *’The Republic’*, as he inherited his forms and ideas from Socrates who was his teacher [33]. Again, Plato’s philosophy declares that there are two worlds: the temporary physical world and the eternal spiritual world [30, 31].

As has already been mentioned, the purpose of this study is to identify the educational philosophical implications of Idealism throughout schools in a city in the United Arab Emirates from the perspectives of mathematics teachers. This sub-section will be presented in the form of the main idealistic educational implications: educational values, curriculum, school functions, the role of teachers, and teaching methods. Additionally, because education is an essential priority and extremely important in the United Arab Emirates, a brief look at the educational system in the United Arab Emirates is the main contribution throughout this subsection, which will first begin with educational values.

### Educational values

According to Fallah et al. [34], idealists consider the human mind as the major substance for understanding the world. Ideals form the vital aim of life and education, connecting conception and schema through the mind [35]. In terms of understanding, the senses are as important as the mind [34]. The idealists’ beliefs indicate that schools perceive knowledge as a liberated unit secluded from sensual experience and understanding, with individuals’ self-realization of who they are and the purpose of such being [36]. Idealistic educational aims focus on training as well as activating the human mind and ignoring physical things [34]. The primary subject matters schools offer to include mathematics, philosophy, art studies, and history based on the origins of idealistic values [34].

The origins of idealistic values remain perpetual and permanent in a particular spiritual natural force, and such everlasting values exist in spiritual entities or mysticism [37]. Goodness and beauty are consistent with the absolute nature of goodness and absolute beauty created by the Al-Mighty Creator (as many people believe) [37]. Because they are significant measures of the stable natural order, their values are absolute and fixed [37]. In enhancing human mind development and achieving individual spiritual growth, educational institutions need to concentrate on individual autonomy and accountability, self-realization, self-control, intellectual activities, and moral judgments as some of the aims of education [34, 37]. Such educational values and objectives require skillful, competent, and capable teachers who are proficient at fulfilling students’ educational/learning needs and help in developing spiritual values as indicated by the idealistic aims of education.

As an imperative idealistic educational implication, the aims of education must guide students’ minds toward accurate or right desires by continuing the communication between consciousness and visualization in rational thinking [38]. The ultimate morality individuals must strive for would be ’happiness’, which leads them to be sophisticated since morals and ethics can be attained and achieved through education [39, 40]. Nations must aim at training human beings to accurate nature to lead their beliefs towards a moral system, because education can develop human beings and pupils or students [22, 41, 42].

’Self-realization’ is the ultimate aim of education in Idealism, and in order to accomplish that state, teachers need to teach and guide their students’ learning styles and methods to be aware of their capacities for bravery and to emulate them [41, 42]. In Idealism, the aims of education include—training for a holy life, improvement of willpower, self-realization, preservation, promotion, the transformation of cultural heritage, mounting harmony in diversity, and the development of spiritual values [42, 43].

The development of spiritual values requires learners to be taught skills such as communication, debate, dialogue, conversation, and discussion in order to gain wisdom about what is attractive, correct, ethical, and honorable [40]. Teachers, as their guides, instruct their learners to become natural and appreciate reflective investigations to progress, advance themselves, and attain character development [16, 19]. The development of self and character in Idealism is another major aim of education that might lead learners to achieve self-actualization [44]. This could be accomplished provided that teachers organize and establish education for the growth of learners’ spiritually, ethically, and morally [42, 43]. In the direction of improving individuals in general and students in particular, the idealistic educational philosophy provided teachers with several remarkable teaching methods.

### Teaching methods

The teaching methods in Idealism are all-inclusive and holistic, since universal character development and self-realization are strengthened by discovering the essence of oneself [18]. The idealists believe that wisdom can be learned and established when rational ethical dispositions, in addition to personal reflections and beliefs, are shown to students and improved. Therefore, it is appropriate at this point to become acquainted with the most significant methods of teaching in Idealism.

In Idealism, the most significant teaching methods are lecturing, discourses, and dialogues [18]. Such methods are accomplished when teachers prepare and choose a topic to clarify and impart idealistic knowledge to the students [41]. They begin by asking students questions (even the right questions) associated with a given issue or subject, and then, students reply to their teachers’ questions [45]. Teachers offer their students feedback through which learners can improve their learning and themselves based on their teachers’ feedback [11, 18, 45]. Idealistic teachers use a teacher-centered method in which learners are passive members, which may create a slight problem with their understanding of the topic [11, 22, 34, 41]. According to Idealism, debates, questions, answers, and dialogues may be effective teaching techniques for helping learners search, analyze, and probe while learning [3, 46, 47].

Learning will most likely happen through dialogue and discussions once teachers motivate learners to activate their thinking capabilities and brainstorm to extract notions and connotations from their minds in school [3, 11]. Schools should be aware of effective teaching methods in respect of individual and spiritual values and through learning about the local setting [48]. Lecturing could be a significant teaching method in schools, including other approaches, to convey actual data to learners, which helps them load their minds with certain truths [49, 50]. Schools ought to implement these methods of examining and synthesizing to solve problems, which is viewed as a traditional philosophy of education [48–50].

As a traditional philosophy of education, Idealism observes teachers as central role models for their learners [42]. Learners must assume and imitate their teachers so that they can become good residents and citizens [43]. To do so, lecturing as an important method of teaching would facilitate learners’ imitation of their teachers and other leading personalities with good virtues, values, leadership, and morals in society [18, 37]. The lecturing method entails materials and contents being transported or reflected by learners while listening to and following their teachers [18, 43]. On the other hand, teachers must apply the inspired moral aptitudes of their students by providing them with chances to comprehend, scrutinize, combine, create, and use knowledge for being and behavior [37, 42, 43]. The implementation of idealistic teaching methods requires an idealistic curriculum as a source of teaching, learning, and assessment.

### Curriculum

The curriculum ought to be established by taking into account the main assumptions of aesthetic and spiritual nature [16, 18]. The spiritual nature of the greater entities such as the family, the state, society, the universe, and eternity would prevail as a significant part of the school curriculum [51]. The school curriculum should contain subjects necessary for understanding the spiritual and honorable improvements of every individual [19, 37]. These subjects that all students must study may well provide them with a cultural heritage that must be transmitted from one generation to another with essential human values, ethics, and moral principles (Elias & Merriam, 1980). The subjects must be stable, consistent, constant, and determined for all learners to develop fully as conscious human beings with the highest level of consciousness and coherence with humanity, ethics, social, cultural, and moral principles [37, 51, 52].

All learners must be provided with subject matters that should be kept constant or consistent across time and space to preserve the core of humanity with all possible moral principles [18, 44]. The idealistic school curriculum must concentrate on human minds as the supreme imperative human structure that can make rational decisions while being able to judge acceptable social values and norms [52, 53]. Children’s abilities and refining their brainpower with a higher mental state of awareness and consciousness might be attained through studying liberal arts and reading by all learners who must study the same subject matters in school, particularly mathematics [34]. Mathematics is considered an important subject matter schools offer in order to impart human minds, in addition to school clubs and classroom activities as extracurricular events [34, 49]. Furthermore, the curriculum, which is based on the idea of the divine nature of humans (men and women), leads to an idea of the nature of the larger units of society, the world, and eternity [33]. The curriculum, which is essential for understanding mental and moral growth, must comprise subjects that provide learners with an ethos and ought to be given to all [18]. Besides, subjects must be continuous for all learners, in addition to ideas as well as concepts [37, 52].

Concepts are organized through lectures, discussions, and dialogues by teachers. A school is a place in which the competencies of reasonable and logical thinking, reasoning, and assessing youngsters are gradually transported, reflected, and improved by progressive teachers. Consequently, wealthy spiritual ethics, values, ideologies, dogmas, and principles would be assimilated through an idealistic curriculum [51, 52].

Schools belong to society as a whole and play an important role in sustaining the cherished cultural heritage, since education aims to preserve, mature, and convey the cultural heritage to future generations [37, 51, 52]. The curriculum, according to Idealism as an educational philosophy, entails certain values and principles [16]. For example, first: the principle of pursuing some qualities such as reality, goodness, and attractiveness. Second, the principle of somatic development and the principle of cognitive, affective, and psychomotor developmental domains are also some values that the curriculum entails [18, 37, 51, 52]. The idealistic educational philosophical domains are derived from the major principles of Idealism, and schools play a significant role in activating these principles as a major part of school functions.

### School functions

Idealist philosophers, such as Plato, Augustine, Descartes, Berkeley, Hume, Kant, Hegel, and Royce had a tremendous impact on the notion of schools as a means of promoting knowledge and preserving social norms and values [14]. School functions are based on idealistic educational beliefs about observing schools as miniature societies that must provide students with stability and sincere learning experiences by teaching them a democratic way of life with ethical and moral values [18]. Schools are homes where ideas are verified, implemented, and simplified [54]. School functions also entail creating remarkable learning environments through which teachers can lead learners to the ultimate reality as much as possible, attaining their fullest potential and guiding them toward their highest reasonable role [55]. Wise teachers regarded as model characters within efficient and expressive schools may have such skills to be effective and operative regarding their pupils’ moral characters [18]. The teachers’ task ought to be to wisely generate an enjoyable learning environment for students [55, 56].

The schools’ interpretations of observed truths entail the human mind as perfect and precise rather than a traditional physical experience [55]. Another primary school function is knowledge and information transmission from one generation to the next, so that popular culture can be preserved through teaching [54]. As an essential curriculum within schools, subject matters must be pleasant and inspire students to develop cooperation, obedience, and respect for others [18]. Idealistic schools work on applying recommendations and directions while taking individual differences into account [34]. Idealistic educators believe that authorized examinations are the finest technique to evaluate pupils’ achievements. Schools could apply punishment simply to regulate learners’ conduct [34, 49]. An additional key feature of school functions based on the educational philosophy of Idealism is the role of teachers.

### Role of teachers

Teachers are the central aspect of the education process, as they are the ideal role models for their pupils, both spiritually and ethically. Teachers must concentrate on curricular activities as essential parts of the school curriculum [49]. The connection between educators and learners should be an official relationship observed by schools and teachers who assess their pupils in light of precise measurements administered by a governing body and the teachers themselves [43, 49, 57].

Teachers themselves are one of the essential constituents, as no other component of the school system is more vital than teachers, of whom idealists have high expectations [18, 49]. Teachers who must shine in their awareness and human understanding of students’ needs and aptitudes should be brilliant to be examples and role models for their learners properly and knowledgeably [43]. To do so, teachers need to create and demonstrate moral merit inserted in their deeds and principles, in addition to using and enjoying outstanding creative competencies through providing the minds of their pupils with opportunities to study, scrutinize, integrate, examine, amalgamate, and put knowledge into their conduct and into their way of life [11, 22, 41, 43, 49]. Hence, it could be determined that teachers have the leading role in learning environments based on the educational teachings of Idealism.

Based on the educational teachings of Idealism, teachers play a dominant role in the pedagogical process [18]. They constantly evaluate their pupils’ learning by asking them precise questions regarding the subject materials they transmit to students through lecturing [15]. Lecturing in Idealism, along with dialogues, are the central and most important teaching methods [15, 34] as has already been mentioned. The teacher’s role is an icon and a representative of good character, a knowledge principal responsible for the pedagogical process, whereas pupils are obstinate receivers and reflectors who learn and embrace the subject matters and materials through their teachers. In Idealism, teachers are responsible for choosing the suitable materials and curricula to deliver them to their learners [11].

In conclusion, idealistic educational implications include, first, the nature of existence, implying that all existing things in the universe are in the soul, spirit, or mind. Second, the subject matters are represented by the preceding generations, who transmit, impart, and inspire ‘Wisdom’ to the next generations. Third, teaching methods help the mental capabilities of pupils to be implanted, motivated, and inspired by the teachers, who then have significant roles. The roles of teachers as a fourth idealistic educational implication implies transmitting social and cultural heritage from one generation to another and being role models before their students spiritually and culturally.

Fifth, the roles of students entail that they are acknowledged as receivers and reflectors who learn and embrace essential values, norms, and principles through skill repetitions and examinations, and they have the autonomy to think immaterially or abstractly. Sixth, the roles of schools are viewed idealistically as mental growth environments in which absolute, unchangeable, consistent, fixed, and idealistic values must be taught to students. Additionally, schools are places where knowledge must be gained through the mind and discovery through reasoning. Idealism’s educational implications, principles, and values ensure human values in debates, dialogues, lectures, and discussions. The values and objectives of the Ministry of Education Strategic Plan 2017–2021 in the United Arab Emirates also ensure human values in the discussion, tolerance, moderation, peace, co-existence, compassion, and volunteering, in addition to ensuring inclusive quality education, including pre-school education [58, 59]. The researchers reviewed some literature focusing only on the five idealistic educational implications that have been mentioned. The next subsection of this part of the current research study includes some related previous studies.

### Previous studies

This subsection of previous studies includes five pertinent topics of concern, starting the most recent. Gordon and White [3], Freedman [60] and Cobben [61] presented specific idealistic educational implications. First, the aim of education in Idealism is the preservation, enrichment, and transmission of culture. Second, education should play a significant role in the improvement of culture and in helping students to become coherent as well as reasonable. Third, education in Idealism refers to making good and appropriate acquaintances between students and teachers.

Momany et al. [62] explored elementary teachers’ perspectives regarding the educational philosophical implications of Idealism as an educational theory throughout public schools in Jordan. Their study findings showed that, with a mean of (2.13), elementary teachers in Jordan negatively regarded the idealistic educational philosophy being implemented throughout public schools. With a mean of (2.3), the content domain placed first, while with a mean of (1.9), the teachers’ domain placed last. In addition, their study findings showed that, with a mean of (2.13), the general perceptions of elementary teachers in terms of executing idealistic educational philosophical principles were consistent across public elementary schools in Jordan [62].

Alharahsha [63] targeted physical education teachers to determine the prevailing philosophy from their perspectives in the directorate of education in Mafraq. Alharahsha used a descriptive technique to build and prepare a questionnaire consisting of thirty-nine statements distributed among the idealistic, realistic, pragmatic, and naturalistic philosophical domains. The study sample consisted of (77) male and female physical education teachers. The study findings revealed that the prevailing or dominant philosophy was the idealistic educational philosophy, which ranked first with a mean of 4.18 [63]. The findings also indicated no statistically significant differences due to such independent variables as gender, educational level, years of experience, and directorate, except for the Pragmatic educational philosophy, in favor of females, and the Eastern Badia Directorate as the dominant teaching philosophical belief [63].

Al-Mowadiyah [64] studied the dominant teaching philosophical beliefs of basic-level English teachers (teaching in grades 1 through 6) and their relation to their classroom practices in the Hashemite Kingdom of Jordan. She used a survey questionnaire consisting of (28) philosophical beliefs and (28) teaching practices that basic-level English language teachers pursue. The study sample consisted of 22 male teachers and 63 female teachers. The instrument contained philosophical beliefs that represented the main four educational philosophies, pioneered by Idealism as an educational philosophy. Al-Mowadiyah [64] concluded that the idealistic educational philosophy teaching practices from the perspectives of English teachers were to a moderate degree with a mean of (3.61). The findings indicated that the educational implementations included the aim of education with a mean score of (4.56) ranking the highest, while the role of teachers with a mean score of (2.94) ranking the lowest [64]. Furthermore, there was a significant positive correlation between English teachers’ philosophical beliefs and their teaching practices. Significant statistical differences did not occur due to gender, but there was a significant statistical difference in favor of five years of experience or above [64].

Gaidori [65] piloted a study on the philosophy of education in Syria concerning Kantian Idealism and its pedagogical dimensions, whose purpose was to identify the conceptions of ideal philosophical educational areas; moral values, roles of teachers, the role of students, curriculum, and school activities required to improve human personality. The content analysis method was employed as a qualitative research method [65]. The results indicated Kantian philosophical beliefs were connected to some educational domains, such as moral values being absolute and mental; education aims to improve existing societal values. Immanuel Kant believed that teachers must be role models for their learners and create pupils in accordance with ethical values [65]. Other major results showed that the school curriculum ought to put emphasis on the spiritual nature of individuals. Additionally, the results confirmed that schooling in general, according to Kantian Idealism, must contribute to developing the logical thinking skills of learners in addition to increasing their perceptions [65].

For the purpose of this research study, these educational values, school curriculum, school functions, roles of teachers, and teaching methods will be studied and examined to reveal to what extent teachers implement them throughout schools in the United Arab Emirates from college students’ perspectives. According to [58, 59, 66], the Ministry of Education in the United Arab Emirates incorporated teaching methods that focused on, in addition to students’ self-learning abilities, other beneficial educational values through the education system in the United Arab Emirates.

### Educational system in the United Arab Emirates

Holistic, Ideal, and Progressive Education has been a top priority in the United Arab Emirates ever since the country’s inception as an independent state. This prioritization is emulated in the contemporary strategic plan for (2017–2021). The purpose of this strategic education plan is to increase the high school graduation percentage as educational rules emulate the nation’s main concern of being equivalent to high achieving nations [58, 67]. The UAE’s first president, Sheikh Zayed Bin Sultan Al Nahyan, may he rest in peace, projected education as a significant constituent of economic upgrading. He believed that the utmost use of wealth would be to invest in the people of the country in building educated and skilled generations. Sheikh Zayed trusted that, by the standard of their education, individuals could prosper and succeed. The nation follows an exceedingly operative globalization strategy which has been developed to be an outstanding international education pivot worldwide, mentally and morally [67, 68].

The moral education program, which has a wide and comprehensive view of moral development within a philosophical understanding of what it means to be moral in the United Arab Emirates, is exceptionally ambitious and determined and a major part of teachers’ roles. Moral Education was officially launched in 2017 as a subject in public as well as private schools across the nation for grades 1–12 [59]. A major role for teachers throughout schools in the United Arab Emirates would be the need to attain a harmony or a neutral stance on argumentative matters and questions so that they inspire their students to engage in evidential and disputable discussions rather than being a source of authority. Teachers also need to share various practices and learning experiences by conducting and involving themselves in action research. In school settings, action research enables teachers as professional practitioners to work together with study mechanisms that help them search for solutions, challenges, and explanations for mutual problems encountered in schools. Teachers may take advantage of the practice of contemplating and planning by using action research studies. This process may help teachers appreciate their profession well and generate competent understanding as they endeavor continuous improvement [59, 67–69]. Continuous improvements in education occur throughout the Emirati schools through appropriate strategic plans within the Ministry of Education.

### Ministry of education strategic plan 2017–2021 in the UAE

"Innovative education for a knowledge-based, pioneering, and global society" [70] is the vision of the Ministry of Education in the United Arab Emirates. In the United Arab Emirates, the Ministry of Education’s mission is to:

Develop an innovative education system for knowledge and a globally competitive society that includes all age groups to meet future labor market demand by ensuring the quality of the Ministry of Education outputs and the provision of the best services for internal and external customers [70].

The values and objectives of the Ministry of Education in the UAE include: first, citizenship and responsibility, which involves enhancing national citizenship and social responsibility. Second, the principles and values of Islam in terms of ensuring human values in discussions, having tolerance, attitude of moderation, seeking peace, and volunteering to contribute to the society. Third, individual and institutional commitment and transparency that embrace committing to professional and transparent performance at personal, social and institutional levels. Fourth, equality and justice by committing to community partnership and accountability in the education process. Fifth, participation and accountability by ensuring equal educational opportunities for all. Moreover, the sixth, science, technology, and innovation by encouraging a society driven by science, technology, and innovation [67, 70, 71].

The Ministry of Education’s strategic objectives furthermore include seven strategic objectives for schools. They are, first, ensuring inclusive, quality education, including pre-school education [70, 71]. The second is achieving excellent leadership and educational efficiency. The third is to ensure quality, efficiency, and good governance of educational and institutional performance, including the delivery of teaching [70, 71]. The fourth is about ensuring safe, conducive, and challenging learning environments. The fifth is related to attracting and preparing students to enroll in higher education both internally and externally, considering labor market needs [67–71]. The sixth one is strengthening scientific research and innovation capacity by following quality, efficiency, and transparency standards [67–71]. The seventh strategic objective is related to providing quality, efficient, and transparent administrative services according to quality, efficiency, and transparency standards [67–71]. Furthermore, the eighth strategic objective of the Ministry of Education in the UAE is to establish a culture of innovation in an institutional working environment [67, 68, 71].

The school education system was formally initiated in the 1960s in the Emirate of Abu Dhabi. However, the most constructive story came into existence in 1971, upon the declaration of the United Arab Emirates Federation, and during the same year, the Ministry of Education was established. Public education services are provided free for all. In 2012, the UAE Administration authorized a new mandate making education a requirement and a fundamental right for every citizen [67–71].

The Ministry of Education has been promptly advancing to complete the process of essential growth and modification in the environment of developing and planning a contemporary educational philosophy [67–71]. The Ministry started with the introduction of the Emirati schools that originated from one of the finest international educational systems. The Ministry also introduced modern curricula for innovation and creativity to prepare a patriotic generation of citizens who are proficient in addition to maintaining modern skills. The Ministry of Education in the UAE envisions a future where education builds a generation that is attentive, aware, and mindful of life’s necessities and demands [67–71].

## Methodology

This section presents the study population, sample, instrument, reliability, and validity of the study questionnaire with 32 items. The researchers implemented these items, which embody the main idealistic educational philosophical principles, in order to collect data from the study sample.

### Study population and sample

The study population consisted of all mathematics teachers teaching in private and public schools in the United Arab Emirates. Multi-stage random sampling was used to select one emirate, Abu Dhabi, out of seven emirates in the UAE and one city out of three cities (Abu Dhabi, Al Dhafra, and Al Ain) in Abu Dhabi. The study sample consisted of 82 randomly selected mathematics teachers (46 male and 36 female) teaching in different public (47) and private (35) schools in the city of Al Ain, Abu Dhabi, UAE, in the fall semester of 2020. The sampling was random, as the researchers had no control over who would participate or not participate in the study. Rather it was dependent on the participants’ choice to participate or not when they received the survey links from the teachers’ professional groups in social media or school administrations. The teachers were teaching in different school cycles and had diverse teaching experiences (Table 1).

**Table 1:** Distribution of the teachers’ sample according to demographic characteristics (N = 82).

Demographic Background		Number of Participants	Percentage
Gender	Male	46	56.1%
	Female	36	43.9%
School Type	Public	47	57.3%
	Private	35	42.7%
School Cycle	Cycle 1	7	08.5%
	Cycle 2	26	31.7%
	Cycle 3	49	59.8%
Teaching Experience	1–5 Years	18	22.0%
	6–10 Years	11	13.4%
	11–15 Years	15	18.3%
	More than 15 years	38	46.3%

### Study instrument

A prior designed questionnaire from Momany et al. [62] was utilized as the instrument for data collection. The questionnaire was designed to explore the philosophical notion of Idealism, covering the areas of curriculum, educational values, school functions, the role of the teacher, and teaching methods. The number of items in different domains is presented in Table 2.

**Table 2:** Distribution of questions among the survey 5 domains (adopted from Momany et al. [62]).

Domain	Items
Curriculum (Cronbach alpha = 0.762)	1. The mind/soul is the most important human organ that the Emirati school curriculum must focus on. 2. The curriculum which is taught to pupils must provide subject matters that should be kept constant for all. 3. Through discussions and dialogue, the teacher focuses on brainstorming to extract ideas and meanings from the minds of students. 4. All students study the same courses within the school. 5. Mathematics is the subject matter that school offers in order to educate the human mind. 6. Extracurricular activities such as school clubs and classroom activities are taken into account by the school.
Educational Values (Cronbach alpha = 0.683)	1. The mind/soul is the primary source of human understanding. 2. Ideals form the ultimate goal in education and life. 3. Senses are no less important than the mind in terms of understanding. 4. The school views knowledge as an independent entity far from the sensual experience. 5. Educational objectives concentrate on exercising the human mind while ignoring physical entities. 6. Extracurricular activities such as school clubs and classroom activities are taken into account by the school.
School Functions (Cronbach alpha = 0.786)	1. The school views facts perceived by the human mind are more accurate than direct sensual experience. 2. The role of the school is to transfer knowledge from one generation to another. 3. The school views subject matter as the core curriculum. 4. The Emirati school maintains popular culture through teaching. 5. The school motivates learners to become cooperative, obedient, and respect others. 6. The school works on implementing suggestions and instructions. 7. Individual differences are taken into consideration by the school. 8. The official examinations are the best way to measure students’ achievements. 9. The school uses punishment in order to adjust students’ behavior.
Roles of the Teacher (Cronbach alpha = 0.735)	1. Teachers in the United Arab Emirates focus on curricular activities that are parts of school curricula. 2. The teacher is the main core of the education process. 3. The teacher is the ideal role model before his/her students mentally as well as morally. 4. The relationship between teachers and students is looked upon as an official perceived by the school. 5. Teachers evaluate their students in light of accurate measurements governed by the governing body which is the Ministry of Education. 6. Teachers evaluate their students in light of accurate measurements governed by the teachers themselves.
Teaching Methods (Cronbach alpha = 0.674)	1. Through discussions and dialogue, the teacher focuses on brainstorming to extract ideas and meanings from the minds of students. 2. The school is concerned to teach students methods as to respect spiritual values and individual values through studying the local environment. 3. Teachers use lecturing as a teaching method to transform real information to their pupils which helps in storing their minds with definite facts. 4. Teachers use such teaching methods as dialogue, discussions, and mental activities in order to solve problems. 5. The school uses such teaching methods as analyzing as well as synthesizing to solve problems.

The construction of the tool was based on the theoretical and practical notion of Idealism and its essential characteristics in an education system in general and the educational values, norms, and principles in the UAE. The selection and finalization of the items in the questionnaire were done in collaboration with the researchers to adapt the original tool in the context of UAE. The item selection at the beginning was done by one researcher, and he shared the questionnaire with other researchers for accuracy and clarity in language, suitability of the items to collect relevant information from the participants, and connection of the items with the main research problem of Idealism.

The researchers carefully assessed both the validity and reliability of the study instrument to ensure its soundness and consistency. Specifically, a total of 4 experts that included 2 faculty members from the United Arab Emirates University, and 2 faculty members from Yarmouk University, reviewed the questionnaire to determine its relevance and suitability for the objective of the study. Their input was considered, and revisions and suggestions made by the validation panel of experts were incorporated into the research instrument. The reliability coefficient of the 32 Likert-type questionnaires was examined with Cronbach’s alpha, which was found to be 0.937, which was high and acceptable. The reliability coefficients for each sub-domain were examined and they were all above 0.600 (See Table 2). The sub-scale curriculum, school function, and role of teachers were reliable (Cronbach’s alphas were greater than 0.7), whereas the sub-scale educational values and teaching methods were marginally reliable, as the reliability coefficient Cronbach’s alphas were below 0.7 but greater than 0.6 [72].

### Data collection

At first, one of the authors applied for ethical approval of the study from the United Arab Emirates University, Al Ain, Abu Dhabi, UAE. The study was approved by the Research Ethics Committee of the United Arab Emirates University with protocol number ERS_2021_7340. Written consent from the participants was obtained as a part of their participation in the study when they signed into the online questionnaire during the data collection process. The data was collected on a voluntary basis via an online survey of mathematics teachers teaching at both public and private schools in the city of Al Ain, in the Emirate of Abu Dhabi. The online version of the survey was distributed to the potential participants through social media and personal contacts of the researchers to the schools. Informed consent was included in the first part of the questionnaire where the participants were informed of the purpose, the time that it may take to respond to the questionnaire, their voluntary participation, the use of data, the safety and privacy of their personal information, and their right to withdraw from the study. The data collection took place during the first school semester of the 2020 academic year.

### Analysis and interpretation

The questionnaire data was transferred to IBM SPSS 26 for coding and analysis. The data had five group variables–curriculum, value, school function, teacher role, and teaching. A composite score value for each group variable was computed by averaging the items under each group. The group composite values were examined for normality. After deciding the normality of each group variable, one sample t-tests were applied to examine the teachers’ views about Idealism in each item and also the composite value as a whole. A mid-value of three (from the Likert-scale) was used as a test item. Then, group comparisons were performed for gender, school type, years of teaching experience, and school cycles either by t-test or one-way ANOVA. For t-tests, we examined the assumptions for the suitability of the tests with continuous data, random sampling, homogeneity of variance in each group, and normality of variables. Likewise, the data were independent for each grouping variable, assumed equal variances, and had a normal distribution of the test variables. Finally, bivariate correlation among the group variables was performed. A generalized linear model (GLM) was applied to examine if there was a significant impact of demographic and other group variables on the teaching practice of mathematics teachers. We considered the demographic information, curriculum, education value, school function and role of teachers as independent variables and the composite score for teaching methods as the dependent variable for GLM. The statistical findings were described and further interpreted in the discussion section with respect to relevant literature.

## Results

**Fig 1:**
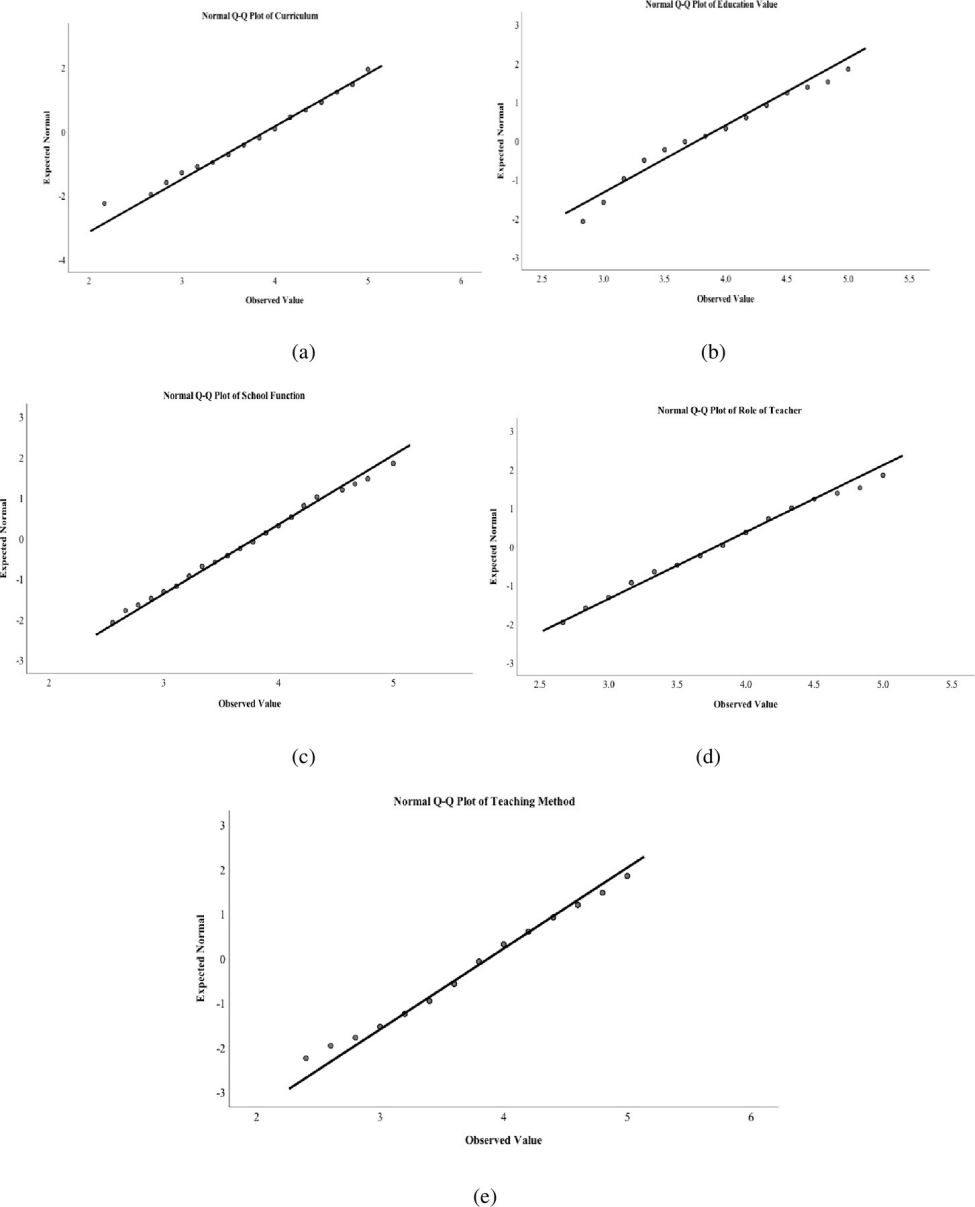
Q-Q plots for the distribution of teacher perception on curriculum (a), education values (b), school function (c), teacher roles (d), and methods of teaching (e).

The data from the 32 items and 82 mathematics teachers were grouped into five different categories related to teachers’ perception of the curriculum, educational values, school function, teachers’ role, and teaching methods. Their composite average values were computed as continuous variables. In order to decide whether to use parametric or non-parametric tests to discuss each of these categories, a test of normality was performed with the Kolmogorov-Smirnov and Shapiro-Wilk tests. The normality test results showed that all five categories were normally distributed at a 0.01 level of significance after the removal of four outliers (Table 3). In addition, the Q-Q plot showed that the data values for these categories were close to the diagonal line. Therefore, we assumed the five continuous composite variables were almost normally distributed (Fig 1). In this way, we decided to use parametric tests based on the normality of these categories of continuous scale variables with a 78 sample size by removing four outliers in the data.

**Table 3:** Test of normality for the five categories of continuous scale variables.

	Kolmogorov-Smirnov			Shapiro-Wilk		
	Statistic	df	Sig.	Statistic	df	Sig.
Curriculum	.112	78	.018	.974	78	.112
Edu. Value	.093	78	.089	.974	78	.111
School Function	.066	78	.200	.982	78	.317
Role of Teacher	.084	78	.200	.973	78	.094
Teaching Method	.153	78	.000	.969	78	.053

### Analysis of categories and items (one-sample t-tests)

A one-sample t-test with a test value of three (mid-value) was performed to examine participants’ responses to the categories and related items in terms of their agreements or disagreements. The results of the one-sample t-test for the *curriculum* showed that the participants had a positive view (agreement) with item 1 (The mind/soul is the most important human organ that the Emirati school curriculum must focus on.), which was statistically significant and positive (Mean = 4.08, SDV = 0.818, and p = 0.000 < 0.05). Similarly, they had positive views on the rest of the other items, all statistically significant (p < 0.05). The overall perceptions of the curriculum was positive (agreement) (Mean = 3.89, SDV = 0.61, and p = 0.000 < 0.05) (Table 4).

**Table 4:** One-sample statistics (curriculum and related items, test value = 3).

	N	Mean	Std. Deviation	Std. Error Mean	Mean Diff.	Sig. (2-tail)
1. The mind/soul is the most important human organ that the Emirati school curriculum must focus on.	78	4.08	.818	.093	1.077	.000
2. The curriculum which is taught to pupils must provide subject matters that should be kept constant for all.	78	3.44	1.234	.140	.436	.003
3. Through discussions and dialogue, the teacher focuses on brainstorming to extract ideas and meanings from the minds of students.	78	4.22	.638	.072	1.218	.000
4. All students study the same courses within the school.	78	3.56	1.202	.136	.564	.000
5. Mathematics is the subject matter that school offers in order to educate the human mind.	78	4.22	.750	.085	1.218	.000
6. Extracurricular activities such as school clubs and classroom activities are taken into account by the school.	78	3.87	1.144	.129	.872	.000
Curriculum	78	3.89	.610	.069	.897	.000

The results of the one-sample t-test for *educational values* (Table 5) showed that the participants had a positive view (agreement) with item 2 (The mind/soul is the primary source for human understanding.), which was statistically significant (Mean = 4.13, SDV = 0.78, and p = 0.000 < 0.05). Similarly, they had positive views on the items "Ideals form the ultimate goal in education and life", "Senses are no less important than the mind in terms of understanding", and "Extracurricular activities such as school clubs and classroom activities are taken into account by the school" that were all statistically significant (p < 0.05). However, the participants had a neutral view on items "The school views knowledge as an independent entity far from sensual experience" and "Educational objectives concentrate on exercising the human mind while ignoring physical entities" (p > 0.05). The overall perception of the participants on educational value was positive and statistically significant (Mean = 3.77, SDV = 0.58, and p = 0.000 < 0.05) (Table 5).

**Table 5:** One-sample statistics (educational values and related items, test value = 3).

	N	Mean	Std. Deviation	Std. Error Mean	Mean Diff.	Sig. (2-tail)
1. The mind/soul is the primary source of human understanding.	78	4.13	.78	.090	1.128	.000
2. Ideals form the ultimate goal in education and life.	78	4.32	.66	.074	1.321	.000
3. Senses are no less important than the mind in terms of understanding.	78	4.42	.68	.076	1.423	.000
4. The school views knowledge as an independent entity far from the sensual experience.	78	3.10	1.21	.137	.103	.457
5. Educational objectives concentrate on exercising the human mind while ignoring physical entities.	78	3.06	1.12	.127	.064	.615
6. Extracurricular activities such as school clubs and classroom activities are taken into account by the school.	78	3.55	1.28	.144	.551	.000
Edu Value	78	3.77	.58	.065	.765	.000

The results of the one-sample t-test for *school function* (Table 6) showed that the participants had a positive view (agreement) with the item "The school views facts perceived by the human mind as more accurate than direct sensual experience", which was statistically significant (Mean = 3.73, SDV = 0.95, and p = 0.000 < 0.05). Similarly, they had positive views on the rest of the items regarding the role of teachers, which were all statistically significant (p < 0.05). The overall perception of the participants on school functions was positive and statistically significant (Mean = 3.79, SDV = 0.58, and p = 0.000 < 0.05) (Table 6).

**Table 6:** One-sample statistics (school function and related items, test value = 3).

Sl. # Items	N	Mean	Std. Dev.	df	Sig. (2-tailed)
1. The school views facts perceived by the human mind are more accurate than direct sensual experience.	78	3.73	.95	.107	.000
2. The role of school is to transfer knowledge from one generation to another.	78	3.90	.89	.101	.000
3. The school views subject matter as the core curriculum	78	3.68	.90	.102	.000
4. The Emirati school maintains popular culture through teaching.	78	4.31	.71	.080	.000
5. The school motivates learners to become cooperative, obedient, and respect others.	78	4.42	.81	.092	.000
6. The school works on implementing suggestions and instructions.	78	3.91	.94	.107	.000
7. Individual differences are taken into consideration by the school.	78	4.10	.99	.112	.000
8. The official examinations are the best way to measure students’ achievements.	78	3.47	1.26	.142	.001
9. The school uses punishment in order to adjust students’ behavior.	78	2.64	1.41	.159	.027
School Function	78	3.79	.58	.066	.000

The results of the one-sample t-test for the *role of teachers* (Table 7) showed that the participants had a positive view (agreement) with the item "Teachers in the United Arab Emirates focus on curricular activities that are part of school curricula", which was statistically significant (Mean = 3.86, SDV = 0.75, and p = 0.000 < 0.05). Similarly, they had positive views on items 12, 16, 20, 29, and 30, which were all statistically significant (p < 0.05). The overall perceptions of the participants on the teacher’s role was positive and statistically significant (Mean = 3.78, SDV = 0.58, and p = 0.000 < 0.05) (Table 7).

**Table 7:** One-sample statistics (role of teachers and related items, test value = 3).

	N	Mean	Std. Deviation	Std. Error Mean	Mean Diff.	Sig. (2-tail)
1. Teachers in the United Arab Emirates focus on curricular activities that are parts of school curricula.	78	3.86	.751	.085	.859	.000
2. The teacher is the main core of the education process.	78	3.46	1.245	.141	.462	.002
3. Teacher is the ideal role model before his/her students mentally as well as morally.	78	4.56	.636	.072	1.564	.000
4. The relationship between teachers and students is looked upon as an official perceived by the school.	78	3.42	.947	.107	.423	.000
5. Teachers evaluate their students in light of accurate measurements governed by the governing body which is the Ministry of Education.	78	4.04	.797	.090	1.038	.000
6. Teachers evaluate their students in light of accurate measurements governed by the teachers themselves.	78	3.32	1.294	.147	.321	.032
Role of Teacher	78	3.78	.58	.065	.778	.000

The results of the one-sample t-test for *teaching methods* (Table 8) showed that the participants had a positive view (agreement) with item 15, which was statistically significant (Mean = 4.22, SDV = 0.64, and p = 0.000 < 0.05). Similarly, they had positive views on items 21, 26, and 28 that were all statistically significant (p < 0.05). However, the participants had a neutral view on item 25 (p > 0.05). The overall perception of the participants on teaching methods was positive and statistically significant (Mean = 3.87, SDV = 0.55, and p = 0.000 < 0.05) (Table 8).

**Table 8:** One-sample statistics (teaching methods and related items, test value = 3).

	N	Mean	Std. Deviation	Std. Error Mean	Mean Diff.	Sig. (2-tail)
1. Through discussions and dialogue, the teacher focuses on br ainstorming to extract ideas and meanings from the minds of students.	78	4.22	.64	.072	1.218	.000
2. The school is concerned to teach students methods as to respect spiritual values and individual values through studying the local environment.	78	4.12	.97	.109	1.115	.000
3. Teachers use lecturing as a teaching method to transform real information to their pupils which helps in storing their minds with definite facts.	78	2.95	1.29	.146	-.051	.726
4. Teachers use such teaching methods as dialogue, discussions, and mental activities in order to solve problems.	78	3.94	.84	.095	.936	.000
5. The school uses such teaching methods as analyzing as well as synthesizing to solve problems.	78	4.15	.85	.097	1.154	.000
Teaching Method	78	3.87	.55	.062	.874	.000

### Comparison of groups

The results of the t-test for the comparison of the means of each of these categories with the participants’ gender (male and female) were performed (Table 9). The distribution of the five variables (curriculum, educational values, school functions, role of teachers, and teaching methods) were normally distributed at a 0.05 level of significance (Table 3, Shapiro-Wilk test), and we presented the results for equality of variances assumed from Levene’s test (which were not significant at a 0.05 level of significance) while comparing them with gender. The results of the t-test showed no statistically significant difference between the male and female teachers concerning their perception of curriculum, educational values, school functions, role teachers, and teaching methods as per the philosophy of idealism at a 0.05 level of significance (Table 9). The effect size measured with Cohen’s d ranged from 0.53 for teaching methods to 0.62 for curriculum, indicating a wider range of gender differences in teachers’ perspectives on curriculum from the viewpoint of idealism.

**Table 9:** Comparison of male and female teachers concerning the five categories.

	Gender	N	Mean	STDEV	Mean Diff.	Sig. (2-tail)
Curriculum	Male	45	3.80	.67	-.24	.08
	Female	33	4.04	.48		
Edu Value	Male	45	3.73	.62	-.08	.53
	Female	33	3.81	.52		
School Function	Male	45	3.75	.64	-.12	.38
	Female	33	3.87	.50		
Role of Teacher	Male	45	3.83	.60	.13	.33
	Female	33	3.70	.54		
Teaching Method	Male	45	3.84	.57	-.08	.52
	Male	33	3.92	.53		

The results of the t-test for the comparison of the means of each of the categories with the participants’ school type (private and public) were performed (Table 10). The distribution of the five variables (curriculum, educational values, school functions, role of teachers, and teaching methods) were normally distributed at a 0.05 level of significance (Table 3, Shapiro-Wilk Test). We presented the results for equality of variances assumed from Levene’s test (which were not significant at the 0.05 level of significance) while comparing them with school types. The results of the t-test showed no statistically significant difference between the teachers of private and public schools concerning their perception of curriculum, educational values, school functions, role of teachers, and teaching methods as per the philosophy of idealism at the 0.05 level of significance (Table 10). The effect size measured with Cohen’s d ranged from 0.53 for teaching methods to 0.63 for school type indicating a wider range of difference between opinions of public and private school teachers’ perspectives on the curriculum from the viewpoint of idealism.

**Table 10:** Comparison of private and public school teachers with respect to the five categories.

	School Type	N	Mean	SDEV	Mean Diff.	Sig. (2-tail)
Curriculum	Public	45	3.91	.55	.03	.87
	Private	33	3.88	.69		
Edu Value	Public	45	3.76	.55	-.01	.97
	Private	33	3.77	.61		
School Function	Public	45	3.84	.51	.10	.48
	Private	33	3.74	.68		
Role of Teacher	Public	45	3.73	.46	-.11	.39
	Private	33	3.84	.71		
Teaching Method	Public	45	3.88	.49	.02	.85
	Private	33	3.86	.64		

The results of a one-way ANOVA comparing the means of each of these categories with participants’ teaching experience were performed (Table 11). The distribution of the five variables (curriculum, educational values, school functions, the role of teachers, and teaching methods) were normally distributed at 0.05 level of significance (Table 3, Shapiro-Wilk Test). We presented the results for the homogeneity of variances based on means from Levene’s test (which were not significant at the 0.05 level of significance) while comparing them across teachers with different years of experiences. The results of the ANOVA showed that there was no statistically significant difference between the teachers of four groups with different years of experience with respect to their perceptions of curriculum, educational values, school functions, the role teachers, and teaching methods as per the philosophy of idealism at the 0.05 level of significance (Table 11). The measure of ANOVA effect size with Eta-squared was the highest for the role of teachers (0.102) and the lowest for school functions (0.005).

**Table 11:** Comparison of teachers with their teaching experience with respect to the five categories.

Variables	Comparison	Sum of Squares	df	Mean Sq.	F	Sig.
Curriculum	Between Groups	2.453	3	.818	2.337	.081
	Within Groups	25.893	74	.350		
	Total	28.346	77			
Edu Value	Between Groups	.613	3	.204	.608	.612
	Within Groups	24.856	74	.336		
	Total	25.469	77			
School Function	Between Groups	.033	3	.011	.031	.993
	Within Groups	26.199	74	.354		
	Total	26.233	77			
Role of Teacher	Between Groups	2.518	3	.839	2.678	.053
	Within Groups	23.186	74	.313		
	Total	25.704	77			
Teaching Method	Between Groups	.611	3	.204	.662	.578
	Within Groups	22.758	74	.308		
	Total	23.369	77			

The results of a one-way ANOVA for the comparison of the means of each of these categories with participants’ teaching school cycles were performed (Table 12). The distribution of the five variables (curriculum, educational values, school functions, role of teachers, and teaching methods) were normally distributed at a 0.05 level of significance (Table 3, Shapiro-Wilk Test). Then we presented the results for homogeneity of variances based on means from Levene’s test (which were not significant at the 0.05 level of significance) while comparing them across teachers with cycles (teaching grades). The results of the ANOVA showed that there was no statistically significant difference between the teachers of the three groups (cycles of teaching) with respect to their perception of the curriculum, the role of teachers, and teaching methods as per the philosophy of idealism at a 0.05 level of significance. However, they were significantly different across the teaching cycles with respect to educational values and school functions at a 0.05 level of significance. The post-hoc analysis showed that the difference between teachers teaching in cycles one and two was statistically significantly different with respect to educational values. Likewise, the post-hoc test for school function showed that teachers of cycles one and two and one and three were significantly different at the 0.05 level of significance (Tables 12 and 13). The effect size measured by Eta-squared was the highest for the education values (0.073) and the lowest for curriculum (0.013).

**Table 12:** Comparison of teachers with teaching cycles with respect to the five categories.

Dep. Variables		Sum of Sq.	df	Mean Sq.		F	Sig.
Curriculum	Between Groups	.794	2	.397		1.081	.344
	Within Groups	27.552	75	.367			
	Total	28.346	77				
Edu Value	Between Groups	2.988	2	1.494		4.985	.009
	Within Groups	22.480	75	.300			
	Total	25.469	77				
School Function	Between Groups	2.438	2	1.219		3.842	.026
	Within Groups	23.795	75	.317			
	Total	26.233	77				
Role of Teacher	Between Groups	.361	2	.180		.534	.589
	Within Groups	25.343	75	.338			
	Total	25.704	77				
Teaching Method	Between Groups	1.764	2	.882	3	.063	.053
	Within Groups	21.604	75	.288			
	Total	23.369	77				

**Table 13:** Post comparison of teachers with teaching cycles with respect to educational values and school functions.

Dep. Variable	School Cycle	School Cycle	Mean Diff.	Std. Err.	Sig. (2-tail)
Edu Value	1	2	-.77564*	.24796	.008
		3	-.57609	.23764	.053
	2	1	.77564*	.24796	.008
		3	.19955	.13433	.425
	3	1	.57609	.23764	.053
		2	-.19955	.13433	.425
School Function	1	2	-.67806*	.25511	.029
		3	-.65298*	.24449	.028
	2	1	.67806*	.25511	.029
		3	.02508	.13820	1.000
	3	1	.65298*	.24449	.028
		2	-.02508	.13820	1.000

A bivariate correlation analysis was performed to examine which pairs among the five categories have highly significant correlations (Table 14). The results showed that teachers’ perception of the curriculum had a high correlation with educational values (r = 0.78) and school function (0.76); and a moderately high correlation with teaching methods (r = 0.70) and the role of teachers (r = 0.61). There was a high correlation between participants’ perceptions of educational values and school functions (r = 0.80) and curriculum (r = 0.78). Similarly, there was a high correlation between school function and teaching methods (r = 0.813) and a moderately high correlation with the role of teachers (r = 0.72). The role of teachers had a low correlation with teaching methods (r = 0.65). All of these correlations were statistically significant at a 0.01 level of significance (Table 14).

**Table 14:** Bivariate correlation analysis of the five categories.

Variables			Curriculum Edu Value	School Function	Role of Teacher Teach.	Method
Curriculum	Pearson Correlation	1	.784**	.759**	.610**	.704**
	Sig. (2-tailed)		.000	.000	.000	.000
	N		78	78	78	78
Edu Value	Pearson Correlation		1	.801**	.656**	.664**
	Sig. (2-tailed)			.000	.000	.000
	N			78	78	78
School Function	Pearson Correlation			1	.718**	.813**
	Sig. (2-tailed)				.000	.000
	N				78	78
Role of Teacher	Pearson Correlation				1	.651**
	Sig. (2-tailed)					.000
	N					78
Teach. Method	Pearson Correlation					1
	Sig. (2-tailed)					
	N					78

** Correlation is significant at the 0.01 level (2-tailed).

A Generalized Linear Model (GLM) fitting was performed with teaching methods as a dependent variable and other categories (curriculum, educational values, school function, and role of teacher) as independent covariates and gender, school type, teaching experience, and school cycle as independent factor variables (Table 15). The results showed that none of the categorical independent variables significantly impacted teaching methods at a 0.05 level of significance. Similarly, there was no statistically significant impact of educational value and perception of teacher roles on teaching methods (p > 0.05). However, there was a statistically significant positive impact of curriculum and school function on the methods of teaching (B_curriculum_ = 0.234, p = 0.029 < 0.05; B_school_function_ = 0.62, p = 0.000 < 0.05) (Table 15).

**Table 15:** Generalized Linear Model fitting for the dependent variable of teaching method with that of independent covariates and categorical variables.

			95% Wald Conf. Interval.		Hypothesis Test		
Parameter	B	Std. Err.	Lower	Upper	Wald Chi-Square	df	Sig.
(Intercept)	.811	.2922	.239	1.384	7.708	1	.005
Gender: Male	.011	.0797	-.145	.167	.019	1	.890
Gender: Female	0a	.	.	.	.		
School Type: Private	.013	.0747	-.133	.160	.031	1	.860
School Type: Public	0a	.	.	.	.		
Experience: 1	.135	.1216	-.103	.374	1.235	1	.266
Experience: 2	.129	.1323	-.130	.388	.952	1	.329
Experience: 3	-.002	.1013	-.201	.196	.000	1	.983
Experience: 4	0a	.	.	.	.		
School Cycle: 1	-.157	.1655	-.481	.168	.895	1	.344
School Cycle: 2	.051	.1001	-.145	.247	.260	1	.610
School Cycle: 3	0a	.	.	.	.		
Curriculum	.234	.1073	.024	.445	4.766	1	.029
Edu Value	-.162	.1177	-.393	.069	1.897	1	.168
School Function	.620	.1235	.377	.862	25.148	1	.000
Role of Teacher	.091	.0998	-.104	.287	.839	1	.360

Dependent Variable: Teaching Method

Model: (Intercept), @1gender, @2Typeofschool, @3YearsofExperience, @4SchoolCycle, Curriculum, Edu Value, School Function, Role of Teacher

## Discussion

Results from the analysis of data (concerning research question 1: (1) To what extent do mathematics teachers believe that the educational philosophical implications of Idealism as an educational theory throughout schools in the United Arab Emirates are implemented?) showed that the overall perception of the participants on the curriculum was positive. That means mathematics teachers in the UAE highly value the mind/soul connection to the mathematics curriculum with the relevant, well-ordered, fundamental subject matter being the same for all, and the use of brainstorming in teaching-learning tasks integrating extra activities. The mathematics teachers’ beliefs about mathematics as associated with mind and soul align with what Plato considered a long time ago, that it trains the mind for logic and takes the human soul toward ultimate truth that may be away from the sensible world [73]. Likewise, they also demonstrated positive belief towards educational values in a statistically significant way. For them (participants), mathematics has great educational values, both ethically and morally. In this sense, their views might contradict the objective reality of mathematics that is modeled by a human being to solve problems, and it is not an object of ethics rather than utility as a way to see its value [74].

The value-associated beliefs reflected mathematics teachers’ beliefs that the mind/soul is the primary source of understanding the world. With the ideals as the ultimate goal of education, schools should promote knowledge as an ideal domain independent of external perceptual reality, and education in general and mathematics, in particular, should embrace the human mind as the principal factor for all knowledge. The Emirati mathematics teachers demonstrated an ideal view of school function in a positive way, which was statistically significant. That means the mathematics teachers in the UAE embrace the ideal that the mind plays a major role in grasping accurate knowledge rather than just sense-dependent information. Idealism is well embraced in the school system with social and cultural values, spirituality, self-realization, and self-control for personal and human development [75]. School transfers knowledge across generations. Core knowledge that is unchanged is valuable for school to transfer and preserve popular culture to develop loyal, obedient, and respectful citizens. The mathematics teachers in Al Ain city have a high regard for the ideal roles of teachers, as their views toward the idealistic role of teachers are positive and statistically significant. As idealist teachers, they prefer to be specialists for their pupils and they wish for cultural preservation for generations through schooling [75]. That means they believe teachers should be the central aspects of the education process who should control teaching-learning as a role model and evaluate students’ performance in a formal way through authority (e.g., Ministry of Education). Assessment should not only focus on subject matter, but also on ethics and morality while conducting such assessments [3]. They highly value discussion and dialogue to enrich students’ minds and spirituality. They consider teacher-directed instruction as a method to disseminate knowledge from mind to mind or soul to soul through lectures and dialogues that enrich thinking, reasoning, and problem-solving.

With reference to findings for research question two (Are there any significant statistical differences between mathematics teachers’ responses due to their gender, type of school, years of experience, and school cycle on all questionnaire items?) mathematics teachers in the UAE seem to be equivocal concerning their perceptions of the curriculum, educational values, school functions, role teachers, and teaching methods as per the philosophy of idealism irrespective of gender, school types, and teaching experience. That means both male and female mathematics teachers embrace idealistic views in teaching mathematics. The findings are consistent with the views of Shahid, Knight, and McNeil [22, 41, 42]. The mathematics teachers of different cycles had no different opinions on the curriculum, the role of teachers, and teaching methods with respect to the teaching cycle. Nonetheless, they have different views on educational values and school functions (Educational Values: cycles 1–2, School Function: cycles 1–2, cycles 1–3). The difference between cycle one and others (cycles two and three) might be due to the fact that cycle one teachers teach at the early childhood and elementary level, whose philosophical views with respect to Idealism could be very different from those who teach at cycles two and three. This shows a difference in teachers’ points of views regarding the ideal role of school to build not only knowledge of the external reality but the consciousness of oneself within one’s mind [4].

With regard to research question three (Is there any significant correlation between teachers’ opinions about curriculum, educational values, school functions, the roles of teachers, and teaching methods to each other?), so far as mathematics teachers’ teaching methods in the UAE are concerned, they have a high correlation (not causation) with school functions. Also, the correlation between the role of teachers and the curriculum seems to be low. That means teachers perception of school function and role of curriculum in their views have a positive correlation [10, 17, 21, 22]. That means teachers, somehow, are able to view the role of school through the curriculum to shape the educational goals or outcomes in mathematics, which is one of the endeavors of idealistic notion of education [76].

Regarding research question 4 (Is there any significant impact of gender, school type, years of experience, cycle of teaching, curriculum perception, value of education, school function, and role of teachers on teaching methods?), the results indicated that gender, school types, years of experience, and the teaching cycles did not have a significant impact on teachers’ conceptions about method of teaching as per the idealism as a philosophy of education. Likewise, there was no significant impact of mathematics teachers’ perceptions of educational values and the role of teachers on teaching methods. This result may be due to self-realization of educational values as teachers to impart moral, ethical, and disciplinary values which are sometimes considered more idealistic than realistic [77]. However, the nature of curriculum and functions of schools as perceived by the mathematics teachers were found to be significant predictors of their self-reported teaching methods. These results signify that schools control the teaching methods. As a result, teachers may not have a significant role in curriculum design, besides teaching or implementing an already designed curricula (e.g., designed by an external authority) [11, 22, 34].

### Implications and conclusion

This study may help educators realize to what extent the idealistic tenets are implemented in the United Arab Emirates and possibly serve as a grounded theory for educators to realize that it is essential to determine and decide on educational beliefs throughout the Emirati schools. Additionally, this study could appeal to mathematics teachers to help them become accustomed to the educational principles of Idealism. The findings of this study may benefit teachers, the school system, and all stakeholders responsible for changing the lives of pupils.

Mathematics teachers in the UAE embrace an idealistic philosophy of curriculum, educational values, school functions, the role of teachers, and overall teaching methods. That means idealistic views still dominate mathematics teachers’ views, impacting their teaching methods. However, schools play a significant role in implementing such views in schools through curriculum and teaching, even if individual teachers may have different opinions (though male and female, private and public school teachers demonstrate similar views). Teaching-mathematics with idealistic principles may preserve the core values of education with a structured and permanent order of curriculum, content, and assessment despite the recent revolution in education with emergent views of STEM/STEAM, and 21^st^-centuary skills within the new education vision of the United Arab Emirates. It can be concluded that mathematics teachers of Al Ain city in the UAE embrace idealistic principles of curriculum, educational values, school and teacher roles, and teaching approach despite reform efforts and the new educational vision of the country.

The study had a major limitation due to a small sample size from one city, Al Ain, that may limit the scope of generalizability of the findings of the study. In this sense, we recommend a study with a larger sample size from all the emirates of the United Arab Emirates. The philosophical views of teachers should be enhanced in teacher education and training programs to help them make judgements about ideal teaching methods examining both from subjective and objective spaces of the world [76, 77]. Mathematics teachers should be developed not only for content and pedagogy, but also for intellectual judgments of their own conscious actions beyond the obvious illusive truths of materialism. They should be trained to think beyond visible to the senses to understand mathematics that is mind-dependent and min-independent [78].

## Supporting information

S1 Data (XLSX)
